# Authoritarianism, Conspiracy Beliefs, Gender and COVID-19: Links Between Individual Differences and Concern About COVID-19, Mask Wearing Behaviors, and the Tendency to Blame China for the Virus

**DOI:** 10.3389/fpsyg.2020.597671

**Published:** 2020-11-26

**Authors:** Eric C. Prichard, Stephen D. Christman

**Affiliations:** ^1^School of Social and Behavioral Sciences, University of Arkansas at Monticello, Monticello, AR, United States; ^2^Department of Psychology, University of Toledo, Toledo, OH, United States

**Keywords:** authoritarianism, conspiracy beliefs, handedness, gender, COVID-19

## Abstract

The present study investigated variables potentially associated with a lack of concern about COVID-19 and belief in the conspiracy theory that China is responsible for the virus. In particular, the study looked at Authoritarianism, Conspiracy Beliefs, gender, and consistency of handedness as predictors of nine Likert-type items gauging attitudes, behavior, and beliefs regarding the virus. Initial analyses showed that Authoritarianism predicted less concern about the impact of the virus on health, less mask wearing, and a stronger belief in China’s responsibility for the illness. Conspiracy Beliefs were associated with a stronger belief in China’s responsibility. Women expressed greater degrees of concern about their own and others’ health and about the financial wellbeing of others. In order to reduce the number of dimensions, and thus the number of tests that could yield a type one error, the nine items were then submitted to a principal components analysis which yielded a “Concern about COVID” factor and a “Blame for China” factor. Authoritarianism is generally associated with less concern about the virus. In addition, men showed less concern about the virus overall than women. Both Authoritarianism and Conspiracy Beliefs accounted for unique variance in blame on China for the virus.

## Introduction

The COVID-19 public health crisis is arguably being driven as much by behavior as by germs. A recent Pew Research Center reported the effect of increasing polarization of attitudes toward the pandemic (Mitchell et al., 2020). Among the findings reported in the study, 38% of respondents believe the seriousness of COVID-19 is being exaggerated, and 36% of all respondents across the study reported believing that it is definitely or probably true that the outbreak was a planned conspiracy. In addition, Mitchell et al. (2020) reported that Americans who rely on Trump and Republican-leaning media outlets for news about the outbreak were more likely to believe the disease is exaggerated, and people who get a lot of their news from social media were more likely to believe that the outbreak was a planned conspiracy.

The last finding is alarming in light of evidence that social media bots are actively being used to spread misinformation about the virus (Ferrara, 2020). Recent investigative journalism has revealed how the collection of social media data can be effectively used by political organizations to target propaganda to specific audiences based on personality and behavioral data collected online (Cadwalladr and Graham-Harrison, 2018). Public knowledge about the type of personality variables that can be targeted may give individual citizens and public policy makers some means of defending themselves against this kind of manipulation. Forewarned is forearmed. Psychological scientists could play an important role in understanding which personality factors contribute to negative health behaviors during the outbreak. If policy makers are going to craft effective messages that encourage people to wear masks and take virus science seriously, they must have an idea of which personality types are more susceptible to ignoring the recommendations of experts and believing in conspiracies.

The present paper focuses on two personality variables and two individual variables that the authors suspect may be related to how seriously people respond to the virus threat. The first is Authoritarianism. Evidence shows that Trump support is associated with Authoritarianism (Choma and Hanoch, 2017), and the Pew Study showed that, among Americans, Trump supporters were more likely to doubt virus science (Mitchell et al., 2020). This raises the possibility that Authoritarianism may be one of the personality traits targeted by the bots investigated by Ferrara (2020). Another potential personality variable of interest is the tendency to endorse Conspiracy Beliefs, a trait that can now be measured using instruments such as that created by Brotherton et al. (2013). As the Pew Study showed, over a third of Americans believe that the virus was planned. It only takes a small number of people who doubt virus science in order to make containment difficult.

The first individual variable investigated in this paper is consistency of handedness. Consistency of handedness has been linked to a number of variables related to gullibility, such as magical ideation and paranormal beliefs (Barnett and Corballis, 2002; Prichard and Christman, 2016). Consistency of handedness has also been linked to Authoritarianism (Christman, 2014; Lyle and Grillo, 2020). Interestingly, it is difficult to predict in which direction handedness might be related to disregard for COVID-19 science. Magical ideation and paranormal beliefs have been linked to inconsistent handedness and left handedness (Barnett and Corballis, 2002; Prichard and Christman, 2016). If consistent handers show less regard for virus science, then it is possible that this relationship is mediated by Authoritarianism. If inconsistent handers show less regard for virus science, then it might be related to gullibility and the tendency to believe conspiracy theories. If there is no handedness effect, these two competing handedness effects may cancel each other out and result in handedness not being of use in predicting COVID-19-specific behavior.

Finally, we included gender as a variable. There is already evidence that men are less likely than women to wear masks (Capraro and Barcelo, 2020), which is notable given evidence that males are more likely to die from COVID-19 than females (Jin et al., 2020). It is worth investigating whether there are other gender differences in behavior and attitudes toward COVID-19.

The present study is empirical and exploratory. It focuses on these four predictors and nine statements regarding attitudes and behaviors related to COVID-19. The first approach was to simply look at the raw correlations between predictors and attitudes and behaviors to the virus. The second approach was to perform an exploratory factor analysis on the items and reduce the total number of factors investigated. This was done in order to reduce the number of tests performed and get an idea of the total contribution of each predictor to the variance in COVID-19-related attitudes and behaviors.

## Methods and Participants

### Participants

Participants were 200 MTurk (*M*_Age_ = 41.58) workers recruited using Amazon MTurk service. Although not a purely representative sample, evidence shows that MTurk can be a reliable source of data when used appropriately (Buhrmester et al., 2011). It also has the advantage of providing a sample that differs from traditional university students. Participants are referred to as workers because they are paid on per survey basis to participate in research. Ninety-one participants were women and 109 were men. Using MTurk’s screening options, we required that all participants report being U.S. High School graduates. This was done to ensure a basic level of English literacy and in part to increase the proportion of participants who are aware of American politics. While political orientation was not measured, Americans have shown a unique link between party affiliation and belief in the seriousness of COVID-19 (Mitchell et al., 2020). Furthermore, Authoritarianism has been associated with support for Donald Trump (Choma and Hanoch, 2017). Thus, among a sample of participants who have an awareness of American politics, we would expect a link between Authoritarianism and a lack of concern about COVID-19. As a secondary screening process, we looked at the reported state of residence for each respondent. Of the participants, 189 reported currently residing in the United States and 11 reported residing in a foreign country. These 11 participants were excluded.

### Procedure

Participants were recruited using MTurk. Participants saw a study titled “Attitudes, Personality, and COVID-19.” The description of the study included a statement saying that the study had been approved by an institutional review board and that by clicking on the link, participants agreed to take on any risks associated with the study. Participants then saw a series of instruments presented through Google Forms. Participants completed all instruments in the same order, which is detailed in the instrument section below. Google Forms allows researchers to make questions required. As a result, we had no missing data. The instruments detailed below were the only ones administered. Upon completion of the survey, participants received a “hit code,” which was a number they were instructed to copy and paste into a field provided on the MTurk webpage. This code was used as verification that the study had been completed. Participants were paid $1 within 72 h of completing the study.

### Instruments

Dependent variables. The dependent measures were created by the authors. After asking participants to report their age, U.S. state of residence, and gender, participants were presented with a series of nine questions broken up into two sections. The first section was titled “Concern About COVID-19” and consisted of five items. There first four items were:

• “How worried and concerned are you about your personal health?”
• “How worried and concerned are you about your financial situation?”
• “How worried and concerned are you about the personal health of your fellow citizens?”
• “How worried and concerned are you about the financial situation of your fellow citizens?”

These items were scored on a seven-point scale from 1 (Not at all concerned) to 7 (Very concerned). The fifth item was “How often do you wear a mask when going out in public?” This item was scored on a seven-point scale from 1 (Always) to 7 (Never). Before analysis, this item was reverse scored.

The second section was titled “Expert Response to COVID-19.” It consisted of the following three items:

• “It is important to listen to and heed the advice given by experts and scientists.”
• “It is important to listen to and heed the advice given by politicians and public figures.”
• “China is directly responsible for the infection rates and death toll in the United States.”

These items were scored on a seven-point scale from 1 (Strongly Disagree) to 7 (Strongly Agree). For the initial analysis, all DVs were treated separately. Later, in order to reduce the number of tests, exploratory factor analysis reduced the dimensions from seven to two. This process will be discussed in the analysis and results section.

Conspiracy beliefs. The Generic Conspiracist Belief Scale (Brotherton et al., 2013) is a fifteen-item scale which measures degree of belief in conspiracies, which the authors define believing in conspiracies when more prosaic explanations are more likely. Example items include: “The government is involved in the murder of innocent citizens and/or well-known public figures, and keeps this a secret,” “Secret organizations communicate with extraterrestrials, but keep this fact from the public,” and “Technology with mind-control capacities is used on people without their knowledge.” The scales consist of 5 point Likert-type items (1—“definitely not true,” 2—“probably not true,” 3—“not sure/cannot decide,” 4—“probably true,” and 5—“definitely true”). For the purposes of making the items more continuous, we changed the five-point scale into a seven-point scale anchored from 1 (Strongly Disagree) to 7 (Strongly Agree). The questions remained the same.

Authoritarianism. Participants were given a ten-item scale intended to assess Authoritarianism. The first part of the scale consisted of the 7-item Authoritarianism subscale of the short version of the Right-Wing Authoritarianism Scale (Rattazzi et al., 2007). The scale consists of items believed to measure the construct of political Authoritarianism (e.g., “Our country desperately needs a mighty leader who will do what has to be done to destroy the radical new ways and sinfulness that are ruining us”). The scale consisted of six-point response scale: Strongly Disagree, Disagree Somewhat, Slightly Disagree, Slightly Agree, Agree Somewhat, Strongly Agree. In the presentation, we made a slight modification. We presented the items as 7-point item anchored from 1 (Strongly Agree) to 7 (Strongly Disagree). In other words, we added an extra level and reversed the polarity of the anchors. In addition to the 7-items they suggested for their Authoritarianism and Submission Subscale (Chronbach’s coefficient = 0.72) we also included the last three Authoritarianism and submission items listed in the appendix. These items were “The only way our country can get through the crisis ahead is to get back to our traditional values, put some tough leader in power, and silence the troublemakers spreading bad ideas,” “Once our government leaders give us the ‘go ahead’, it will be the duty of every patriotic citizen to help stomp out the rot that is poisoning our country from within,” and “What our country really needs is a strong, determined leader who will crush evil, and take us back to our true path.” Once the scale was completed, we reverse scored the items so that a higher score indicated greater Authoritarianism.

Handedness. In order to measure handedness, we used the modified version of the Edinburgh Handedness Inventory (Oldfield, 1971) which was factor analyzed by Christman et al. (2015). It is a 10-item scale which asks participants which hand they prefer, and how strongly they prefer it, for each one of 10 tasks. There are five response options: “Always Right,” “Usually Right,” “No Preference,” “Usually Left,” and “Always Left.” During scoring, “Always Right” and “Always Left” are assigned scores of +10 and −10 respectively. “Usually Right” and “Usually Left” are assigned scores of +5 and −5. No preference is assigned a score of zero. Participants can be divided into “Consistent” and “Inconsistent” groups by summing each person’s score, taking the absolute value of the summed scores, and dividing participants via the median split method. Alternatively, the absolute value of the summed scores, which may range from 0 to 100, can also serve as a continuous measure of consistency of handedness. A score of zero is ambidexterity. A score of 100 is a strong preference for one’s dominant hand. For the analyses below, we used consistency of handedness as a continuous variable.

In Table 1, we list descriptive statistics for all of the variables as well as Cronbach’s alpha for each independent variable. Where a measure of reliability is not applicable, because the variable is a one-item scale, the table says N/A under “Cronbach’s alpha.”

**TABLE 1:** Descriptive statistics for all continuous variables.

**Variable**	**Mean**	***SD***	**Cronbach’s alpha**
Consistency of handedness	80.83	11.17	0.964
Authoritarianism	31.6	20.17	0.976
Conspiracy beliefs	44.53	23.34	0.96
How worried and concerned are you about your personal health?	4.45	1.84	N/A
How worried and concerned are you about your financial situation?	4.26	1.86	N/A
How worried and concerned are you about the personal health of your fellow citizens?	4.90	1.63	N/A
How worried and concerned are you about the financial situation of your fellow citizens?	4.82	1.60	N/A
How often do you wear a mask when going out in public?	5.19	2.08	N/A
It is important to listen to and heed the advice given by experts and scientists	6.12	1.25	N/A
It is important to listen to and heed the advice given by politicians and public figures	3.98	1.66	N/A
China is directly responsible for the infection rates and death toll in the United States	3.33	2.10	N/A

## Analysis and Results

### Factor Reduction and Raw Correlations

The four predictor variables of interest were Conspiracy Beliefs, Authoritarianism, Handedness, and Gender. The first part of the analysis investigated the raw correlations between the predictor variables and the dependent variables. Because the research was exploratory, we did not correct for family-wise error initially. However, after investigating the raw correlation coefficients, we factor analyzed the nine dependent variables and reduced the number of dimensions from nine to two. We also included two of our predictor variables in a set of two regression models. This reduced the number of comparisons to two. Conclusions take both sets of analyses into consideration.

Tables 2, 3 show correlations between predictors and the outcome variables. Table 2 shows correlations between the first three predictor variables and the items from the “Concern About COVID-19” section of the survey. Authoritarianism was significantly negatively associated with concern for others’ health and with self-reported frequency of wearing a mask at the 0.01 level. Authoritarianism was negatively associated with concern for one’s personal health at the 0.05 level. Conspiracy Beliefs were positively associated with concern with one’s personal health at the 0.05 level. During coding, women were given a score of one and men were given a score of two. A negative correlation coefficient means that women show a trait to a greater degree. Women expressed a greater degree of concern about personal health, a greater degree of concern about the health of fellow citizens, a greater degree of concern about the financial well-being of other citizens.

**TABLE 2:** Correlations between predictor variables and “concern about COVID-19” items.

	**How worried and concerned are you about your personal health?**	**How worried and concerned are you about your financial situation?**	**How worried and concerned are you about the personal health of your fellow citizens?**	**How worried and concerned are you about the financial situation of your fellow citizens?**	**How often do you wear a mask when going out in public?**
Authoritarian	−0.177*	0.025	−0.227**	−0.113	−0.228**
AbsHand	0.019	−0.025	−0.012	0.041	0.061
Conspiracy	0.162*	0.091	−0.032	−0.002	−0.13
Gender	−0.156*	0.001	−0.155*	−0.150*	−0.73

**Correlation is significant at the 0.01 level (2-tailed). *Correlation is significant at the 0.05 level (2-tailed).

**TABLE 3:** Correlations between predictor variables and “expert response to COVID-19” items.

	**It is important to listen to and heed the advice given by experts and scientists.**	**It is important to listen to and heed the advice given by politicians and public figures.**	**China is directly responsible for the infection rates and death toll in the United States.**
Authoritarian	−0.261**	0.044	0.349**
AbsHand	0.062	0.03	−0.045
Conspiracy	−0.179*	−0.075	0.282**
Gender	−0.85	−0.002	0.003

**Correlation is significant at the 0.01 level (2-tailed). *Correlation is significant at the 0.05 level (2-tailed).

Table 3 shows correlations between the predictors and items under the “Expert Response to COVID-19” section. Authoritarianism was negatively associated with the belief that it is important to listen to scientists and experts during the crisis, and positively associated with the belief that China is responsible for the outbreak. Conspiracy beliefs showed the same pattern, albeit the correlations were only significant at the 0.05 level. Handedness showed no positive associations with any of the dependent variables. None of the first three predictor variables were correlated in this dataset. Handedness and gender were modestly related, with women reporting a slight tendency toward more consistent handedness, *r* = *0.*144, *p* = 0.042.

The general pattern from the correlation matrix suggests that people with more authoritarian tendencies are less concerned about the virus’s impact on their and others’ health, are less inclined to wear masks, are less inclined to listen to experts, and are more likely to believe China is directly responsible for the virus. People high in Conspiracy Beliefs are more inclined to be concerned about their own health, but also express less trust in experts and greater beliefs in Chinese responsibility for the virus. Women seemed to be more concerned about the impact of the virus on themselves and others, but did not report a greater likelihood of wearing a mask. However, we did nothing to correct for family-wise error rates. In order to reduce the number of comparisons, we engaged in a two-step process. The first step was to perform a principal components analysis of the nine items using SPSS version 25. The analysis offered three dimensions with eigenvalues of greater than one, however, we initially accepted a two-factor solution that clustered the nine items into two dimensions. The reason we accepted the two-factor solution is that the third factor consisted of one item, concern about one’s personal financial situation, which also loaded onto factor one. As will be shown below, when this item is included in factor one, it gives factor one sufficient internal reliability, which is to say a Chronbach’s alpha of greater than 0.70. For that reason, the item was included in factor one. The factor matrix is shown in Table 4. The first factor consists of the items “How worried and concerned are you about your personal health,” “How worried and concerned are you about your financial situation,” “How worried and concerned are you about the personal health of your fellow citizens,” “How worried and concerned are you about the financial situation of your fellow citizens,” “How often do you wear a mask when going out in public,” and “It is important to listen to and heed the advice given by experts and scientists.” A reliability check showed that Cronbach’s alpha = 0.73. This factor was called “Concern about COVID-19.” The second factor consisted of the items “It is important to heed and listen to the advice given by politicians and public figures” and “China is directly responsible for the infection rates and death toll in the United States.” However, a reliability check indicated that Cronbach’s alpha = 0.326, an unacceptable level of reliability. Upon investigation of the initial correlation matrix, we found that blame for China, but not trust of public figures, was related to both Authoritarianism and Conspiracy Beliefs. For that reason, we dropped the item “It is important to heed and listen to the advice given by politicians and public figures” and chose “Blame for China” as factor two.

**TABLE 4:** Factor loading matrix.

	**1**	**2**
How worried and concerned are you about your personal health?	0.724	0.233
How worried and concerned are you about your financial situation?	0.402	0.261
How worried and concerned are you about the personal health of your fellow citizens?	0.853	0.000
How worried and concerned are you about the financial situation of your fellow citizens?	0.563	−0.072
How often do you wear a mask when going out in public?	0.615	−0.289
It is important to listen to and heed the advice given by experts and scientists	0.760	−0.116
It is important to listen to and heed the advice given by politicians and public figures	0.352	0.619
China is directly responsible for the infection rates and death toll in the United States	−0.229	0.812

*Extraction method: principal component analysis.*

### Regression Models

Once we reduced our dependent variables to two factors, we ran two regression models. Each model included Authoritarianism, Conspiracy Beliefs, and gender as predictors. Since the initial correlation matrix found no effect of handedness, it was left out of the final two models. Table 5 shows both regression models. For the first model, the “Concern about COVID-19” factor is the outcome variable. The overall model is significant. Authoritarianism had a significant negative relationship with concern. The more authoritarian attitudes that were endorsed, the less concerned participants were about COVID-19. Gender was also related to concern. Women showed more concern than men. Because both Gender and Authoritarianism were related to concern, we added the interaction term between Gender and Authoritarianism as part of a second step. The interaction term explained little additional variance and did not improve model fit.

**TABLE 5:** Regression models.

	***R***	***R*^2^**	***b***	***SE***	**β**	***T***	***p***
DV one: concern about COVID-19	0.287	0.082					0.001
Authoritarianism			−0.079	0.023	−0.241	−3.516	0.001
Conspiracy belief			0.007	0.020	0.025	0.360	0.719
Gender			−2.009	0.915	−0.152	−2.196	0.029
DV two: blame on China for COVID-19	0.455	0.207					<0.001
Authoritarianism			0.029	0.007	0.277	4.325	<0.001
Conspiracy belief			0.031	0.006	0.347	5.423	<0.001
Gender			0.141	0.270	0.033	0.524	0.601

The second model, also displayed in Table 5, used blame of China for COVID-19 as the dependent variable. The overall model was significant. Furthermore, both Authoritarianism and Conspiracy Beliefs uniquely predicted the tendency to give China more blame for the illness. Because both Conspiracy Beliefs and Authoritarianism were related to blame for China, we added the interaction term between Conspiracy Beliefs and Authoritarianism as part of a second step. The interaction term explained little additional variance and did not improve model fit.

## Discussion

Before attempting any interpretation, it is important to note that the present study is exploratory and should serve as a hypothesis-generating study. It should not be considered a set of hypothetico-deductive tests of *a priori* hypotheses. Some researchers even caution that the use of *p*-values in exploratory research is potentially misleading (e.g., Nosek et al., 2018). While we have elected to keep the *p*-values in the paper, we caution readers to focus primarily on the direction and the size of the effects. The effect sizes should be used to estimate future sample sizes. Finally, the reader should keep in mind that our explanations for the findings are tentative. This must be kept in mind because of both the exploratory and correlational nature of the study.

Among a sample of American high school graduates, Authoritarianism was associated with less overall concern about the virus. Authoritarianism in this context can be defined as feelings of aggression toward people who violate their social norms and unthinking submission to authority. In a recently published paper, Prichard and Christman (2020) used the exact scale which was used in the present study and found that, among Republican primary voters, Authoritarianism was associated with support for Donald Trump. This might explain an odd aspect of the findings. Authoritarianism was also associated with a reduced likelihood of wearing a mask and a greater tendency to blame the virus on China. Conspiracy Beliefs are also related to a greater tendency to blame the virus on China. Interestingly, there was not a significant correlation between Conspiracy Beliefs and Authoritarianism. One potential explanation is that Authoritarianism among American high school graduates is associated with support for Donald Trump, as has been suggested by other studies (Choma and Hanoch, 2017; Prichard and Christman, 2020). Donald Trump has blamed China for the virus (McNeil and Jacobs, 2020) and refused to wear a mask (Blake, 2020). Hence, the trend for authoritarians to be mistrustful of scientists and experts and to refuse to wear a mask might reflect political views as opposed to a general tendency to believe in conspiracy theories. Related recent research offers further reason to suspect that political beliefs may be confounding the negative relationship between Authoritarianism and mask wearing. In a study of the relationships between Life History Orientation and COVID-19-related precautions, Corpuz et al. (2020) reported evidence that political conservatism (which included both social and economic factors) was associated with less endorsement of virus-related precautions and mandatory vaccination. Manson (2020) found that as of April, both Left-wing Authoritarianism and Right-wing Authoritarianism were associated with endorsement of stringent methods to prevent the spread of COVID-19. Our findings would seem to be contradictory, unless enough individuals who score high in authoritarian traits are following the lead of politicians who downplay the virus, such as Donald Trump (Blake, 2020). This raises the question of whether politics have led people who endorse authoritarian views, in particular Right-wing Authoritarians, to shift away from being concerned about the virus. According to this hypothesis, people who score high in Left-wing Authoritarianism would still support stringent anti-virus measures, but Right-wing Authoritarians will have shifted their views. This is a hypothesis that can be tested empirically, and should be of interest to researchers.

In the present study, Authoritarianism and Conspiracy Beliefs each made a separate contribution to explaining the variance associated with the belief that China is responsible for the virus. As stated above, Authoritarians and Conspiracists are not necessarily the same people. Conspiracy Beliefs were not related to the tendency to wear a mask, which might be explained by the fact that Conspiracy Beliefs were positively associated with concern for one’s personal health. Authoritarianism was associated with a lower likelihood of wearing a mask. As such, the effects of inaccurate messaging about the virus may play differently with different people. A conspiracist who believes that China released the virus as part of a plot may be ill informed, but still worried enough to take preventative measures. An authoritarian who admires Donald Trump may blame China because Trump has openly blamed China for the virus (McNeil and Jacobs, 2020), but may also follow Trump’s lead in refusing to wear a mask (Blake, 2020). Future research should replicate the same findings using the measures reported in the present paper. However, we recommend future studies take two additional steps. The first is to measure political orientation. We did not do so in this study, but it would help clarify the link between Trump support, Authoritarianism, and Conspiracy theories. In addition, it would be helpful to have samples which are predominantly from outside of America. The patterns may differ in countries that had different styles of leadership in response to the pandemic. For example, China may also be considered an authoritarian country, but China was able to use this to enforce strict lockdowns (Volpicelli, 2020). In that context, mask wearing and Authoritarianism may be positively related.

Our results did not exactly replicate the findings of Capraro and Barcelo (2020) insofar as men did not express a significantly lower tendency to wear masks. However, men expressed less concern overall regarding COVID-19.

Handedness did not predict any of the outcome variables. There are several possible explanations. One is that, as mentioned earlier, there may be a tendency of inconsistent handers to believe in conspiracies because of gullibility and for consistent handers to believe in conspiracies because of authoritarian tendencies. Another possibility is these particular nine COVID-19-related outcome variables are just not related to handedness and, despite handedness’s usefulness as a predictor of other psychological variables (Prichard et al., 2013), it is not a particularly important variable for the study of these outcomes.

Psychologists have a potentially useful role to play in combating the misinformation that is worsening the impact of the virus. Part of the way to do this is to understand the personality variables and individual differences that predict COVID-19-related behaviors and sensitivities to anti-science messaging. Private firms have long been studying personality for their own purposes (Cadwalladr and Graham-Harrison, 2018). Bots are already targeting people with various kinds of misinformation with the help of data mining (Ferrara, 2020). At a time when COVID-19 is causing harm to peoples’ health, the multiple studies of relationships between personality and COVID-19-related behaviors have the potential to serve as an important firewall against those who would use personality-driven findings to spread misinformation about COVID-19.

## Data Availability Statement

The datasets presented in this study can be found in online repositories. The names of the repository/repositories and accession number(s) can be found below: https://osf.io/qjsr2/?view_only=404917253d4240ed9564ed24b8af6021.

## Ethics Statement

The studies involving human participants were reviewed and approved by University of Arkansas at Monticello Human Subjects IRB. Written informed consent for participation was not required for this study in accordance with the national legislation and the institutional requirements.

## Author Contributions

EP collected and analyzed the data. Both authors wrote approximately 50% of the manuscript.

## Conflict of Interest

The authors declare that the research was conducted in the absence of any commercial or financial relationships that could be construed as a potential conflict of interest.

## References

[B1] Barnett K. J. Corballis M. C. (2002). Ambidexterity and magical ideation. *Laterality* 7 75–84. 10.1080/13576500143000131 15513189

[B2] Blake A. (2020). *Republicans are Practically Begging Trump to Tell People to Wear a Mask. He Still Won’t Listen.* Washington, DC: Washington Post.

[B3] Brotherton R. French C. C. Pickering A. D. (2013). Measuring belief in conspiracy theories: the generic conspiracist beliefs scale. *Front. Psychol.* 4:279. 10.3389/fpsyg.2013.00279 23734136 PMC3659314

[B4] Buhrmester M. Kwang T. Gosling S. D. (2011). Amazon;s mechanical turk: a new source of inexpensive, yet high-quality, data? *Perspect. Psychol. Sci.* 6 3–5. 26162106 10.1177/1745691610393980

[B5] Cadwalladr C. Graham-Harrison E. (2018). Revealed: 50 million Facebook profiles harvested for Cambridge Analytica in major data breach. *Guardian* 17:22.

[B6] Capraro V. Barcelo H. (2020). The effect of messaging and gender on intentions to wear a face covering to slow down COVID-19 transmission. *arXiv* [preprint] 10.31234/osf.io/tg7vz PMC8013666 33821089

[B7] Choma B. L. Hanoch Y. (2017). Cognitive ability and authoritarianism: understanding support for trump and clinton. *Personal. Individ. Differ.* 106 287–291. 10.1016/j.paid.2016.10.054

[B8] Christman S. (2014). Individual differences in personality as a function of degree of handedness: consistent-handers are less sensation seeking, more authoritarian, and more sensitive to disgust. *Laterality: Asymmetries of Body, Brain and Cognition*, 19 354–367. 10.1080/1357650X.2013.838962 24088015

[B9] Christman S. D. Prichard E. C. Corser R. (2015). Factor analysis of the edinburgh handedness inventory: inconsistent handedness yields a two-factor solution. *Brain Cogn.* 98 82–86. 10.1016/j.bandc.2015.06.005 26143558

[B10] Corpuz R. D’Alessandro S. Adeyemo J. Jankowski N. Kandalaft K. (2020). Life history orientation predicts COVID-19 precautions and projected behaviors. *Front. Psychol.* 11:1857. 10.3389/fpsyg.2020.01857 32793087 PMC7393224

[B11] Ferrara E. (2020). # covid-19 on twitter: bots, conspiracies, and social media activism. *arXiv* [preprint], Available at: https://arxiv.org/abs/2004.09531 (accessed July 8, 2020).

[B12] Jin J.-M. Bai P. He W. Wu F. Liu X.-F. Han D.-M. (2020). Gender differences in patients with COVID-19: focus on severity and mortality. *Front. Public Health* 8:152. 10.3389/fpubh.2020.00152 32411652 PMC7201103

[B13] Lyle K. B. Grillo M. C. (2020). Why are consistently-handed individuals more authoritarian? The role of need for cognitive closure, *Laterality*, 25 490–510. 10.1080/1357650X.2020.1765791 32498598

[B14] Manson J. H. (2020). Right-wing authoritarianism, left-wing authoritarianism, and pandemic-mitigation authoritarianism. *Personal. Individ. Differ.* 167:110251. 10.1016/j.paid.2020.110251 32834284 PMC7365073

[B15] McNeil D. G. Jacobs A. (2020). *Blaming China for the Pandemic, Trump Says U.S. Will Leave the W.H.O.* New York, NY: The New York Times.

[B16] Mitchell A. Jurkowitz M. Oliphant J. B. Shearer E. (2020). *Three Months In, Many Americans See Exaggeration, Conspiracy Theories and Partisanship in COVID-19 News.* Washington, DC: Pew Research Center.

[B17] Nosek B. A. Ebersole C. R. DeHaven A. C. Mellor D. T. (2018). The preregistration revolution. *Proc. Natl. Acad. Sci. U.S.A.* 115 2600–2606. 10.1073/pnas.1708274114 29531091 PMC5856500

[B18] Oldfield R. C. (1971). The assessment and analysis of handedness: the Edinburgh inventory. *Neuropsychologia* 9 97–113. 10.1016/0028-3932(71)90067-4 5146491

[B19] Prichard E. Propper R. E. Christman S. D. (2013). Degree of handedness, but not direction, is a systematic predictor of cognitive performance. *Front. Psychol.* 4:9. 10.3389/fpsyg.2013.00009 23386836 PMC3560368

[B20] Prichard E. C. Christman S. D. (2016). Need for cognition moderates paranormal beliefs and magical ideation in inconsistent-handers. *Laterality* 21 228–242. 10.1080/1357650x.2015.1125914 26886152

[B21] Prichard E. C. Christman S. D. (2020). Handedness and the 2016 US Primaries: consistent handedness predicts support for Donald Trump among republicans, but gender predicts support for hillary clinton among democrats. *Laterality* 1–13. 10.1080/1357650X.2020.1810061 32842873

[B22] Rattazzi A. M. M. Bobbio A. Canova L. (2007). A short version of the right-wing authoritariaNism (RWA) Scale. *Personal. Individ. Differ.* 43 1223–1234. 10.1016/j.paid.2007.03.013

[B23] Volpicelli G. (2020). *China has Almost Eliminated Covid-19. What can the World Learn?.* London: Wired UK.

